# Neuro-oncological emergencies requiring critical care intervention

**DOI:** 10.1016/j.clinme.2026.100653

**Published:** 2026-10-06

**Authors:** Joshua A. Kornbluth, Jessica Wallace, David Y. Hwang

**Affiliations:** a Department of Neurology (Neurocritical Care), Tufts Medical Center, Tufts University School of Medicine, Boston, MA, USA; b Department of Neurology, Tufts Medical Center, Tufts University School of Medicine, Boston, MA, USA; c Division of Neurocritical Care, Department of Neurology, University of North Carolina School of Medicine, Chapel Hill, NC, USA

**Keywords:** Brain neoplasms, Neuro-oncology, Intracranial hypertension, Status epilepticus, Venous thromboembolism, Immune effector cell-associated neurotoxicity syndrome

## Abstract

Patients with primary or metastatic brain tumours can deteriorate abruptly, and the acute physician is often the first to respond. This review covers the recognition and initial management of the neurological emergencies most likely to need critical care: raised intracranial pressure from peritumoural oedema and herniation; seizures and status epilepticus, including non-convulsive status epilepticus (NCSE); intracranial haemorrhage; acute hydrocephalus; and venous thromboembolism. It also covers two treatment-related emergencies that the general physician must now recognise: immune effector cell-associated neurotoxicity syndrome after chimeric antigen receptor (CAR) T-cell therapy, and immune checkpoint inhibitor encephalitis. Throughout, the text indicates whether recommendations rest on guidelines and where on observational data. The focus is on recognising time-critical treatment, timely escalation to neurosurgical and neurocritical care services, and the early, values-based discussion of goals of care.

## Highlights

• Give dexamethasone for symptomatic peritumoral oedema; treat suspected herniation on declining consciousness or a new deficit, without waiting for a fixed pupil or imaging, with hyperosmolar therapy and immediate neurosurgical referral.
• Do not prescribe prophylactic anti-seizure medications to seizure-naïve brain-tumour patients; new status epilepticus often signals new structural injury, needs a benzodiazepine followed by early second-line therapy, and may be non-convulsive, requiring EEG.
• Cancer-related intracranial haemorrhage is usually intratumoral or coagulopathic rather than hypertensive; reverse coagulopathy, treat symptomatic oedema and seek a surgical opinion, because nearly half of patients are discharged at least partly independent.
• Anticoagulate established VTE in brain-tumour patients with LMWH or a DOAC; DOACs are at least as safe as LMWH with respect to intracranial bleeding.
• Recognise CAR T-cell ICANS and checkpoint-inhibitor encephalitis early; treat with corticosteroids, escalate, and pair emergency management with an early goals-of-care discussion.

## Introduction

About 14 million people worldwide were diagnosed with cancer in 2014, a figure projected to exceed 20 million by 2030.^1^ Brain metastases are more common than malignant primary central nervous system tumours (annual incidence 8.3 vs approximately 6.6 per 100,000), and just over 12% of patients with metastatic disease have brain metastases at initial staging.^1^ These patients can deteriorate abruptly, and the general physician is often the first to respond. Emergencies may be the result of the tumour and its oedema, from systemic effects of cancer such as coagulopathy and thrombosis from treatment, and these mechanisms often coexist. A seizure, for example, may herald haemorrhage into a tumour.^1^, ^2^ The core emergencies are summarised in Table 1.

**Table 1:** Neuro-oncological emergencies at a glance.

Emergency	Typical presentation	Key initial investigation	Immediate management	Trigger to escalate
**Raised ICP/peritumoral oedema & herniation**	Progressive headache, vomiting, reduced consciousness; declining consciousness or new focal deficit; fixed dilated pupil or new focal deficit if herniating and posturing are late signs	Urgent CT head (do not delay treatment of suspected herniation)	Dexamethasone (loading 10 mg, then 4–8 mg/day if mild, ≥16 mg/day if severe); head up 30°, normocapnia, hyperosmolar therapy (Table 2)	Declining consciousness, new deficit or suspected herniation → immediate neurosurgery/NCC referral
**Seizures/status epilepticus**	Witnessed convulsion, or failure to return to baseline, or fluctuating consciousness (non-convulsive)	Capillary glucose, CT head; EEG if non-convulsive suspected	Benzodiazepine first-line at 5 min (IV lorazepam 0.1 mg/kg or IM midazolam 10 mg); second-line ASM without delay if ongoing; treat NCSE until electrographic seizures stop	Refractory seizures, suspected NCSE → urgent EEG + NCC
**Intracranial haemorrhage**	Sudden headache, focal deficit, depressed consciousness	CT head; coagulation screen, platelets	Reverse coagulopathy, control BP, corticosteroids for symptomatic peritumoral oedema	Surgical evacuation/EVD assessment; impaired consciousness or hydrocephalus
**Acute (obstructive) hydrocephalus**	Headache, vomiting, gaze palsy, declining consciousness; posterior fossa tumour	CT/MRI showing ventricular dilatation	CSF diversion (EVD, ETV or VP shunt) if symptomatic; no prophylactic diversion without ventricular dilatation	Urgent neurosurgery for diversion±tumour resection
**Venous thromboembolism**	Limb swelling (DVT) or breathlessness/chest pain (PE)	Doppler/CTPA; review recent neuroimaging for haemorrhage	Therapeutic anticoagulation (LMWH or DOAC) after bleeding-risk assessment; minimum six months	Massive PE, or anticoagulation contraindicated (consider IVC filter)

ICP, intracranial pressure; NCC, neurocritical care; ASM, anti-seizure medication; EEG, electroencephalography; NCSE, non-convulsive status epilepticus; EVD, external ventricular drain; ETV, endoscopic third ventriculostomy; VP, ventriculoperitoneal; DVT, deep vein thrombosis; PE, pulmonary embolism; CTPA, CT pulmonary angiography; LMWH, low-molecular-weight heparin; DOAC, direct oral anticoagulant; IVC, inferior vena cava; BP, blood pressure; CSF, cerebrospinal fluid; IM, intramuscular; IV, intravenous.

## Raised intracranial pressure: peritumoral oedema and herniation

Vasogenic oedema from blood–brain barrier disruption surrounds most tumours and may exceed the mass effect of the lesion itself.^3^ The brain compensates for a slowly enlarging mass, but once a critical threshold of intracranial compliance is crossed, small further increases in volume can cause rapid rises in intracranial pressure (ICP) and may lead to cerebral herniation. Corticosteroids, preferable dexamethasone, are the mainstay of treatment for symptomatic peritumoral oedema. Evidence is insufficient to recommend them for asymptomatic oedema.^4^ Dexamethasone is preferred for its potency and low mineralocorticoid activity. For brain metastases, the Congress of Neurological Surgeons guideline recommends starting 4–8 mg daily for mild symptoms and 16 mg daily or more for severe symptoms of raised ICP (level 3 evidence). A 10 mg intravenous loading dose is often given, and improvement is expected within 48 h.^3^, ^4^ Dexamethasone reduces vascular endothelial growth factor–mediated barrier permeability and does little for the cytotoxic oedema of infarction.^5^ Toxicity, including hyperglycaemia, myopathy, and gastrointestinal complications, rises with both cumulative dose and duration. In one retrospective series, 75% of patients whose total dose exceeded 400 mg (reached after 25 days at 16 mg) had toxicity, compared with 13% of those who received less. No time window is defined, and one-third of patients developed toxicity within three weeks. Steroids should be tapered as rapidly as clinical response allows.^4^

Herniation should be anticipated rather than awaited. Worsening headache, vomiting, declining consciousness or a new progressive focal deficit should prompt immediate treatment a fixed dilated pupil, abnormal posturing and the Cushing response are late signs and, and treatment should wait neither for them to emerge of for imaging confirmation.^3^ Initial measures are summarised in Table 2. Hyperosmolar therapy with hypertonic saline (HTS) or mannitol are rescue therapies or bridges to a more definitive treatment. Mannitol can leak across the disrupted blood-brain-barrier and worsen vasogenic oedema with repeated doses, and in the absence of tumour-specific trial the choice between HTS and mannitol is extrapolated from other brain injuries.^1^, ^3^, ^6^ Brief hyperventilation can bridge to definitive treatment, but sustained hypocapnia risks cerebral ischaemia.^6^ Definitive control needs neurosurgical interventions such as debulking, decompression or cerebrospinal fluid diversion, so referral should be immediate; steroids and osmotherapy only buy time.^3^

**Table 2:** Emergency management of suspected raised intracranial pressure or herniation in a patient with a brain tumour.

Step	Action	Practical points
**Recognise**	Worsening headache or vomiting, declining consciousness, new or progressing focal deficit	Fixed dilated pupil, posturing and Cushing response are late signs; do not wait for them or for imaging
**Stabilise**	Head of bed 30°, neck neutral; secure the airway; avoid hypoxia and hypotension	Normocapnia (PaCO₂ 35–40 mmHg); maintain CPP ≥60 mmHg and avoid CPP <50 mmHg
**Hyperosmolar rescue**	Mannitol 0.5–1.5 g/kg over 10–20 min, or hypertonic saline (23.4% bolus via central access, or 2–3% infusion)	Monitor sodium, osmolality and fluid balance; wean hypertonic saline gradually; repeated mannitol may worsen vasogenic oedema
**Bridge**	Brief hyperventilation for impending herniation	Short-term only: sustained hypocapnia risks cerebral ischaemia
**Treat oedema**	Dexamethasone 10 mg IV, then 16 mg/day in divided doses	Benefit over hours to days; not a stand-alone rescue treatment
**Definitive**	Immediate neurosurgical and NCC referral	Resection or debulking, decompression, or CSF diversion

Based on references.^1^, ^3^, ^4^, ^6^ CPP, cerebral perfusion pressure; CSF, cerebrospinal fluid; IV, intravenous; NCC, neurocritical care; PaCO₂, arterial partial pressure of carbon dioxide.

## Seizures and status epilepticus

Seizures occur in 35–70% of patients with brain tumours and are the presenting feature in 20–40%.^7^ In a patient with a newly diagnosed tumour who has not had a seizure, prophylactic anti-seizure medications (ASMs) are not recommended as they do not reduce seizure risk (level A); the evidence is likewise insufficient to recommend routine prophylaxis in the perioperative period (level C), though considerable practice variation remains.^7^ When treatment is indicated, a non-enzyme-inducing ASM such as levetiracetam is preferred to minimise interactions with cancer therapy; valproate combined with temozolomide increases haematological toxicity.^7^

Convulsive status epilepticus is a neurological emergency. A benzodiazepine is given once a seizure has lasted 5 min: intravenous lorazepam 0.1 mg/kg (maximum 4 mg, repeated once if needed), or intramuscular midazolam 10 mg without intravenous access. If seizures continue, a second-line ASM (intravenous levetiracetam, valproate or fosphenytoin) follows without delay, within the 20–40-min phase. Critically ill patients may need to move through the phases faster.^8^ among patients with brain metastases who develop status epilepticus, only a minority have a prior seizure history, and the event usually signals new or enlarging metastases or haemorrhage.^9^ Non-convulsive status epilepticus also requires prompt treatment. Ongoing non-convulsive seizures may cause neuronal injury and are associated with higher mortality, particularly when diagnosis is delayed.^10^ It should be suspected in any tumour patient who does not return to baseline after a convulsion or has unexplained, fluctuating consciousness, and confirmed with electroencephalography (EEG), continuous EEG where available.^2^, ^9^, ^10^ In one brain metastasis series, status epilepticus was often refractory, with 30-day mortality of about one-third, so early and neurocritical care involvement is essential.^9^

## Intracranial haemorrhage

Intracranial haemorrhage in patients with cancer arises through distinct mechanisms relative to the general population. In a cohort of 208 patients, intratumoral haemorrhage (61%) and coagulopathy (46%) accounted for most events, while hypertension was rare (5%).^11^ The primary origin of the tumour matters in two ways. Some tumours have a high intrinsic propensity to bleed: haemorrhage has been reported in up to half of melanoma brain metastases in surgical series, and renal cell, thyroid and choriocarcinoma metastases also bleed readily.^12^, ^13^ Others cause many haemorrhages because they are common: melanoma, lung, glioma and breast tumours are the commonest causes of cancer-related intracranial haemorrhage, and leukaemia the commonest haematological cause, through coagulopathy.^11^, ^12^ Melanoma is therefore over-represented through both frequent brain involvement and a high propensity to bleed. Contrast-enhanced imaging, once the patient is stable, helps distinguish an intratumoral bleed from primary intracerebral haemorrhage. Management follows usual haemorrhage principles, reversing coagulopathy and controlling blood pressure, plus corticosteroids for vasogenic oedema around an intratumoral bleed and early neurosurgical assessment.^12^ Thirty-day mortality is ~31%, yet nearly half are discharged at least partly independent, supporting active early management; ventriculostomy placement was associated with better survival.^11^

## Acute hydrocephalus

Posterior fossa tumours, or any that obstruct the ventricular system, can cause acute obstructive hydrocephalus, with headache, vomiting, depressed consciousness and, untreated, herniation. About one-fifth of adults (and up to 80% of children) with posterior fossa tumours have hydrocephalus at presentation, which resolves after resection in ~96% of adults.^14^ Cerebrospinal fluid diversion is indicated for symptomatic hydrocephalus, whereas prophylactic pre-resection diversion is not recommended in the absence of ventricular dilatation.^14^ Options are an external ventricular drain (which also allows ICP monitoring), endoscopic third ventriculostomy, or ventriculoperitoneal shunting, though resection of the obstructing tumour is usually definitive.^2^, ^14^ External drains should be removed as soon as possible, ideally within eight days, to limit infection and shunt dependence.^14^ Upward herniation or intratumoral haemorrhage after pre-resection drainage is uncommon (2.8% in a large paediatric series) but requires neurosurgical oversight.^2^, ^14^

## Venous thromboembolism

Patients with primary brain tumours have among the highest rates of venous thromboembolism (VTE) of any cancer group: 20–30%, and around 30% per year in glioblastoma.^15^ Established VTE should still be treated with systemic anticoagulation. The 2022 International Initiative on Thrombosis and Cancer (ITAC) guidelines state that either low-molecular-weight heparin (LMWH) or a direct oral anticoagulant (DOAC) can be used for VTE in patients with a brain tumour (grade 2A: weak recommendation based on high-quality evidence), for at least six months as in cancer-associated VTE in general (grade 1A).^16^ In a glioma cohort of 277 patients, intracerebral haemorrhage rates during anticoagulation occurred in 2.0% with DOACs and 2.3% with LMWH.^15^ Full-dose anticoagulation is reasonable with platelets >50 × 10^9^/L and no bleeding. For patients with a platelet count <50 × 10^9^/L, decisions and treatment dose must be decided on a case-by-case basis (grade: expert guidance).^16^ After neurosurgery, postoperative prophylaxis with LMWH or unfractionated heparin is recommended (grade 1A).^16^ Inferior vena cava filters are reserved for patients in whom anticoagulation is contraindicated, and with anticoagulation restarted once safe.^16^

## Emergencies of modern oncological therapy

Cellular and immune therapies can cause neurological emergencies that the general physician must now recognise, summarised in Table 3. CAR T-cell therapy can cause immune effector cell-associated neurotoxicity syndrome (ICANS), with a median onset 5–7 days after infusion, usually after cytokine release syndrome. It ranges from tremor and dysgraphia to aphasia, encephalopathy, seizures and cerebral oedema. Encephalopathy is scored with the 10-point Immune effector Cell-associated Encephalopathy (ICE) score, but the ICANS grade is set by the most severe of the ICE score, level of consciousness, seizures, motor findings and raised ICP or cerebral oedema.^17^, ^18^ Corticosteroids are first line treatment; tocilizumab has little penetration into cerebrospinal fluid, does not usually improve ICANS and may worsen it, so it is not recommended without concurrent cytokine-release syndrome.^2^, ^17^, ^18^ Immune checkpoint inhibitors cause neurological immune-related adverse events, including encephalitis, in 1–3% of patients. Along with cardiac events, these carry the highest mortality of any immune-related adverse events (8–14%).^19^ Management is to stop the drug, exclude infection and tumour progression, start high-dose corticosteroids early (for example, intravenous methylprednisolone 1 g daily for 3–5 days) and involve a specialist, escalating to intravenous immunoglobulin or plasma exchange if needed.^2^, ^19^

**Table 3:** Treatment-related neurotoxicity: two emergencies the general physician must recognise.

Feature	CAR T-cell ICANS	Checkpoint-inhibitor neurotoxicity (incl. encephalitis)
**Trigger**	CAR T-cell therapy (often after cytokine release syndrome)	Immune checkpoint inhibitor (anti-PD-1/PD-L1/CTLA-4)
**Typical onset**	Median 5–7 days after infusion (range 1 day to 3–4 weeks)	Usually within first three months of treatment
**Presentation**	Tremor, dysgraphia, inattention → encephalopathy, seizures, cerebral oedema	Encephalitis, meningitis, neuropathy, myositis/myasthenia
**Grading/recognition**	10-point ICE score → ASTCT grade 1–4	CTCAE grading; high index of suspicion
**First-line treatment**	Corticosteroids (±supportive NCC care)	Stop drug; exclude infection and tumour progression; early high-dose corticosteroids; IVIG/PLEX if refractory; specialist referral
**Key pitfall**	Tocilizumab does not usually treat ICANS and may worsen it; avoid without concurrent cytokine release syndrome	Neurological irAEs carry disproportionately high mortality

ICANS, immune effector cell-associated neurotoxicity syndrome; ICE, Immune effector Cell-associated Encephalopathy; ASTCT, American Society for Transplantation and Cellular Therapy; CTCAE, Common Terminology Criteria for Adverse Events; irAE, immune-related adverse event; NCC, neurocritical care; IVIG, intravenous immunoglobulin; PLEX, plasma exchange.

## Conclusion

Neuro-oncological emergencies call for rapid, protocol-driven treatment, but the often-guarded prognosis makes an early conversation about goals of care just as important. An overly pessimistic early prediction after acute brain injury can become self-fulfilling. Where the outcome of the acute event is uncertain, a period of full treatment, at least 72 h, is advisable before any decision to limit treatment, weighed against the course of the underlying cancer and the patient’s values through shared decision making^20^ The acute physician therefore has two jobs from the outset: time-critical treatment, and honest, compassionate conversations about goals of care.

## CRediT authorship contribution statement

**Joshua A. Kornbluth:** Writing – review & editing, Writing – original draft, Conceptualization. **Jessica Wallace:** Writing – review & editing, Conceptualization. **David Y. Hwang:** Writing – review & editing, Conceptualization.

## Declaration of generative AI and AI-assisted technologies in the writing process

During the preparation of this work the author(s) used Claude (Anthropic) to assist with copy editing of the initial manuscript draft. After using this tool, the author(s) reviewed and edited the content as needed and take(s) full responsibility for the content of the publication.

## Funding

This research did not receive any specific grant from funding agencies in the public, commercial, or not-for-profit sectors.

## Declaration of competing interest

The authors declare that they have no known competing financial interests or personal relationships that could have appeared to influence the work reported in this paper.
